# Supplementary material for: Snake C-Type Lectins Potentially Contribute to the Prey Immobilization in Protobothrops mucrosquamatus and Trimeresurus stejnegeri Venoms
Source: Toxins (Basel). 2020 Feb 6;12(2):105. doi: 10.3390/toxins12020105 (PMC7076790; doi:10.3390/toxins12020105)
Supplement: Supplementary file 1 [file toxins-12-00105-s001.pdf]

Snake C-Type Lectins Potentially Contribute to the Prey Immobilization in *Protobothrops mucrosquamatus* and *Trimeresurus stejnegeri* Venoms

Huiwen Tian, Ming Liu, Jiameng Li, Runjia Xu, Chengbo Long, Hao Li, James Mwangi, Qiumin Lu, Chuanbin Shen and Ren Lai

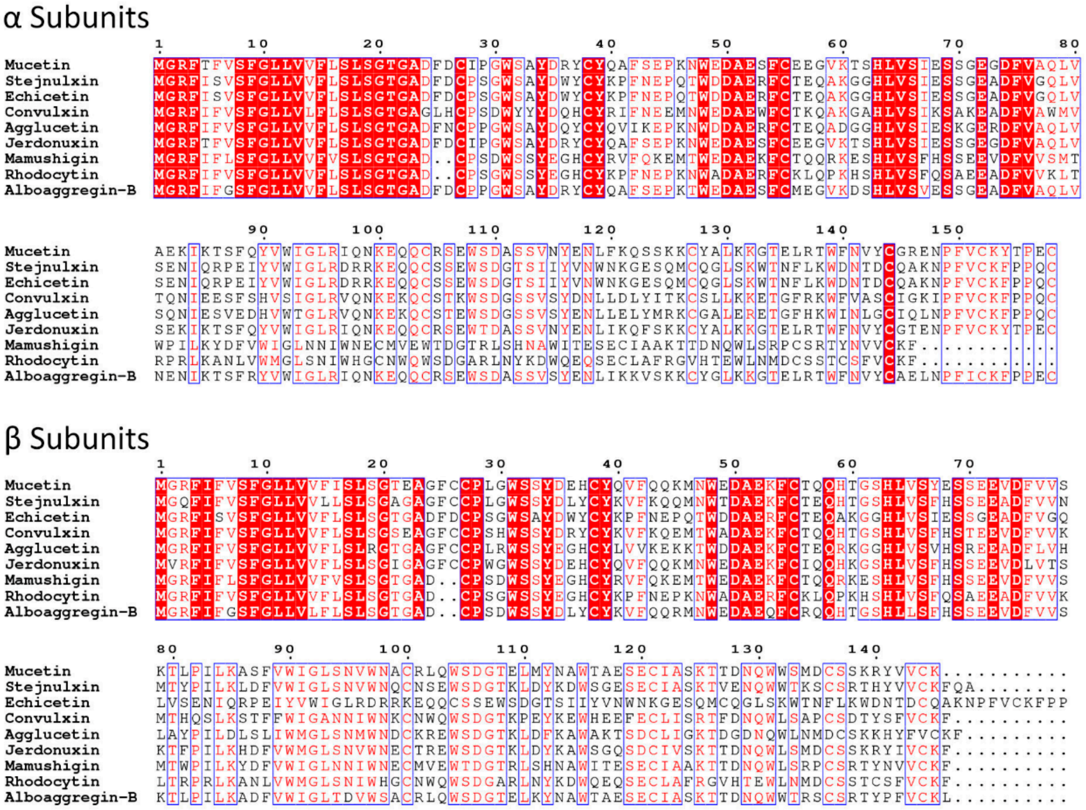

Figure 1. The amino acid sequences of some snake venom C-type lectin-like proteins.

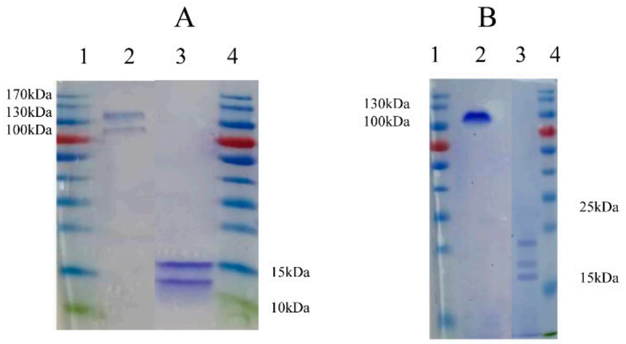

Figure 2. Purity of purified mucetin and stejnulxin as determined by SDS-PAGE and Coomassie blue staining. **A**. The figure shows the SDS-PAGE pattern of purified mucetin: Lanes 1 is from a 10% gel PAGE under non-reducing conditions; lane 4 is from a 12% gel PAGE under reducing conditions. Lanes 1 and 4 are markers. Lanes 2 and 3, purified stejnulxin. **B**. The figure shows the SDS-PAGE pattern of purified stejnulxin: Lanes 1 and 2 are from a 10% gel PAGE under non-reducing conditions;

lane 3 and 4 are from a 12% gel PAGE under reducing conditions.Lanes 1 and 4 are markers. Lanes 2 and 3, purified stejnulxin.
